# Roles of Non-Coding RNAs in Primary Biliary Cholangitis

**DOI:** 10.3389/fmolb.2022.915993

**Published:** 2022-07-08

**Authors:** Yaqin Zhang, Ziying Jiao, Mingwei Chen, Bing Shen, Zongwen Shuai

**Affiliations:** ^1^ Department of Rheumatology, The First Affiliated Hospital of Anhui Medical University, Hefei, China; ^2^ Department of Physiology, School of Basic Medicine of Anhui Medical University, Hefei, China; ^3^ Department of Endocrinology, The First Affiliated Hospital of Anhui Medical University, Hefei, China

**Keywords:** primary biliary cholangitis, non-coding RNA, autoimmunity, ursodeoxycholic acid, biomarker

## Abstract

Primary biliary cholangitis (PBC) is an autoimmune-mediated chronic cholestatic liver disease, fatigue, and skin itching are the most common clinical symptoms. Its main pathological feature is the progressive damage and destruction of bile duct epithelial cells. Non-coding RNA (NcRNA, mainly including microRNA, long non-coding RNA and circular RNA) plays a role in the pathological and biological processes of various diseases, especially autoimmune diseases. Many validated ncRNAs are expected to be biomarkers for the diagnosis or treatment of PBC. This review will elucidate the pathogenesis of PBC and help to identify potential ncRNA biomarkers for PBC.

## Introduction

**FIGURE 1:**
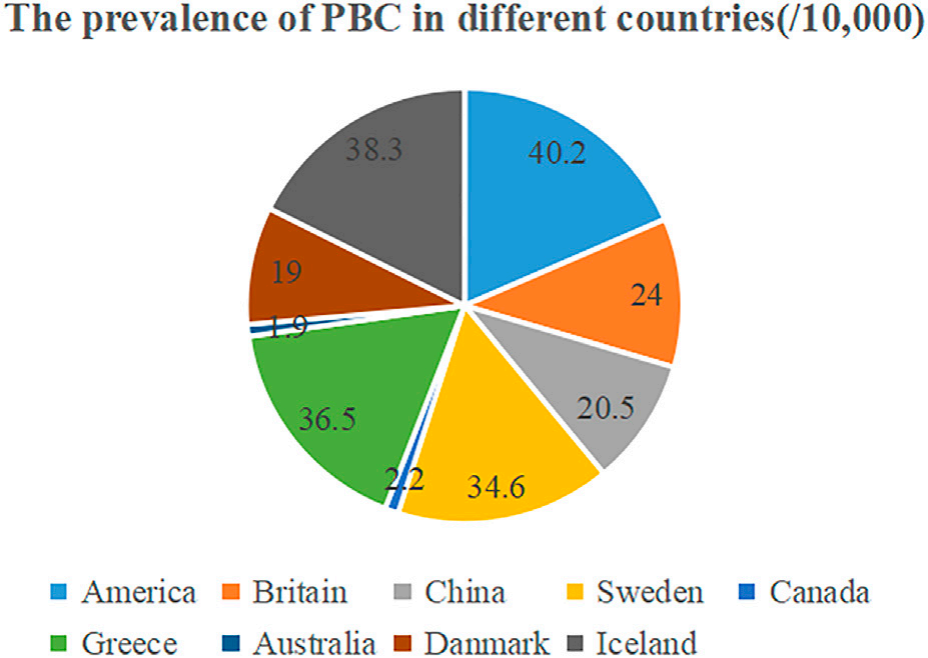
The prevalence of PBC in different countries around the world.

Primary biliary cholangitis (PBC) is a chronic cholestatic liver disease mediated by autoimmunity. Fatigue and pruritus are the most common clinical symptoms, usually preceding the appearance of jaundice by years (Erice et al., 2018; Beuers et al., 2015). The main histological features are chronic progressive damage and destruction of bile duct epithelial cells (non-suppurative cholangitis), portal inflammation (mainly lymphocytes, plasma cells, and eosinophils) and increased fibrosis (Poupon, 2010; Tsuneyama et al., 2017; Pandit and Samant, 2022). PBC is globally distributed and can occur in all races and ethnicities. A recent meta-analysis (Lv et al., 2021) showed that both the incidence and prevalence of PBC are on the rise globally, with an annual incidence rate of 0.23/100,000–5.31/100,000, and a prevalence of 1.91/100,000–40.2/100,000. The highest in North America and Nordic countries (Kim et al., 2000; Koulentaki et al., 2014; Metcalf et al., 1997; Marschall et al., 2019), and the lowest in Canada (Witt-Sullivan et al., 1990) and Australia (Watson et al., 1995). Population-based epidemiological data on PBC are still lacking in China. A recent meta-analysis (Zeng et al., 2019) estimated that the prevalence of PBC in China was 20.5/100,000, ranking second in the Asia-Pacific region after Japan. Figure 1. The cause of PBC is unknown, and may be caused by a highly complex interaction between genetic and environmental factors. There is also extensive evidence that inflammation and immune disorders have an important impact on PBC. The disease mostly affects middle-aged women (i.e., 85%–90% patients onset at 40–60 years old), with female-to-male ratios is about 1:10, while higher mortality was described in men (Gonzalez and Washington, 2018). Moreover, there are also recent literature of increasing in male PBC (Lleo et al., 2016). This may be related to additional environmental exposures, higher prevalence of viral hepatitis, greater awareness of the disease by physicians and patients and unknown gender factors that may modulate immunity (Lleo et al., 2016). The specific reasons need to be further explored.

PBC was also previously known as primary biliary cirrhosis. The name change reflects the fact that cirrhosis only occurs in the late stage, so it is difficult to correctly identify patients with early disease. According to the AASLD (American Association for the Study of Liver Diseases), two of the following three criteria are met to confirm the diagnosis of PBC: 1) biochemical evidence of cholestasis based on alkaline phosphatase (ALP) elevation; 2) anti-mitochondrial antibody (AMA) positivity; 3) histological evidence of intrahepatic destructive cholangitis (Carey et al., 2015). Among them, serum AMA, especially the positive AMA-M2 subtype, has high sensitivity and specificity for the diagnosis of PBC, and are recognized as distinct diagnostic biomarkers. There is also a PBC patient subpopulation (∼5%–10%) who have the same clinical and histological features as classic PBC, but AMAs are negative even with the most sensitive detection methods. For these patients, the diagnosis is mainly based on liver biopsy (Kouroumalis et al., 2018). However, problems can arise in the presence of ascites or infection and are not easily accepted by patients.

The first-line treatment of PBC is the daily administration of ursodeoxycholic acid (UDCA), which improves prognosis in ∼2/3 of patients in early stage of the disease. This means up to 40% of patients have an incomplete response to UDCA, and the long-term survival rate is lower than that of the general population (Parés et al., 2006). UDCA was approved by the United States FDA (Food and Drug Administration) in 2016 as a second-line treatment for patients with primary biliary cholangitis who are unresponsive to UDCA; however, approximately 50% of patients might need additional treatments to reach therapeutic goals. Otherwise, these patients may progress to liver transplantation or even die (Gulamhusein and Hirschfield, 2020). Therefore, identification of novel and promising biomarkers is crucial for PBC early diagnosis and (or) treatment.

Non-coding RNA (NcRNA) refers to a functional RNA molecule that cannot be translated into protein. They can perform their respective biological functions at the RNA level (Wang et al., 2019). Due to increasing development of microarray sequencing techniques, accumulating data have suggested ncRNAs play important roles in regulating autoimmunity and inflammation (Kempinska-Podhorodecka et al., 2017; Young et al., 2017; Wang et al., 2018a). In addition, different cells and tissues have different ncRNA expression profiles (Chen et al., 2016; Katsumi et al., 2016; Zheng et al., 2017a; Erice et al., 2018; Xiang et al., 2019). Erice studies have shown that miR-506 is overexpressed in cholangiocytes of PBC, induces PBC-like characteristics in cholangiocytes and promotes immune activation (Erice et al., 2018). Dai et al. compared miRNAs in renal biopsy samples from patients with class II lupus nephritis (LN) and nephrectomy samples from patients with renal tumors. The results showed that there were 66 differentially regulated miRNAs (36 upregulated and the remaining 30 downregulated) in lupus nephritis patients (Dai et al., 2009). Wu et al. showed that linc0949 and linc0597 were significantly decreased in patients with SLE compared with patients with RA and healthy control subjects. Moreover, linc0949 was positively correlated with SLEDAI-2K scores and negatively correlated with complement component C3 levels (Wu et al., 2015). However, the roles of these specifically expressed ncRNAs in the pathogenesis of PBC have not been fully elucidated.

In the present review, some functional ncRNAs are listed in Table 1, mainly including microRNAs (miRNAs), long non-coding RNAs (lncRNAs), and circular RNAs (circRNAs) (Katsushima et al., 2014; Nakagawa et al., 2017; Wang et al., 2017; Wasik et al., 2017; Afonso et al., 2018; Wasik et al., 2020). We aimed to elucidate the dysregulated ncRNAs in PBC that contribute to the understanding of the pathogenesis of PBC by reviewing all currently published studies. Most importantly, helping to identify those aberrantly expressed ncRNAs in PBC will facilitate the exploration of promising biomarkers for early diagnosis and treatment of PBC.

**TABLE 1:** Aberrant expressed ncRNAs in PBC.

NcRNA	Target	Site	Expression	Signaling	Role	References
**MiRNA**						
miR-506	AE2/InsP3R3	Intrahepatic bile ducts	Up	---	Binds the 3′UTR region of AE2 mRNA, prevents protein translation; Decreased AE2 activity; Impair bile secretion	Erice et al. (2018)
miR-155	SOCS-1	Liver tissues	Up	VDR-miRNA 155-SOCS1 pathway	Sustained inflammatory responses are elicited through the VDR-miRNA155-SOCS1 pathway	Kempinska-Podhorodecka et al. (2017)
miR-139-5p	c-FOS/TNF-α	Serum	Down	NF-κB signaling	Regulated TNF-α and c-FOS.	Katsumi et al. (2016)
		Liver tissues (lymphocytes or hepatocytes)	Up-lymphocytes Down- hepatocytes			
miR-21/miR-150	cMyb/RASGRP1/DNMT1	Serum, liver tissue, and PBMC in AMA (-) patient	Up	---	A feature of anti-mitochondrial antibody-negative PBC.	Wasik et al. (2020)
miR-425	N-Ras	Peripheral blood CD4^+^ T cell	Down	TCR signaling	Downregulation of inflammatory cytokines (IL-2 and IFN-γ)	Nakagawa et al. (2017)
miR-223-3p/miR-21-5p	TGFBR1	Peripheral blood B cells	Down	TGF-β1 signaling	Associated with progression of PBC.	Wang et al. (2017)
miR-34a/miR-132	NRF2	Liver tissues	Down	Oxidative stress	Oxidative stress; Autophagy	Wasik et al. (2017)
miR-21	CDK2AP1	Liver tissues	Up	Regulated necrosis	MiR-21 ablation ameliorates liver damage and necroptosis	Afonso MB, et al., (2018)
miR-92a	IL-17A	Plasma and PBMC	Down	Th17 signaling	Direct regulation of IL-17A	Liang et al. (2016)
miR-181a	BCL-2	Peripheral blood CD4^+^ T cell	Down	TCR signaling	Regulated Th17 cells distribution *via* upregulated BCL-2	Song et al. (2018)
miR-122a/miR-26a	---/EZH2	Liver tissues	Down	Apoptosis/inflammation/oxidative stress/metabolism	Affected cell proliferation, apoptosis, inflammation, oxidative stress, and metabolism	Padgett et al. (2009)
miR-328/miR-299-5p	---		Up			
miR-26a	IL-17A	Th17 cell	Up	---	Regulates IL-17, induces apoptosis and proliferation	Tang (2009)
miR-34a	TGIF2	Peripheral blood	Up	TGF-β1/Smad	Induction of EMT and fibrosis in intrahepatic bile duct epithelium	Pan et al. (2021)
**Circulating miRNAs**						
miR-299-5p	---	Peripheral blood	Up	---	MiR-299-5p was associated with ALP, γ-GT, TBIL and immunoglobulin M levels	Katsushima, et al. (2014)
let-7b	---	PBMC	Down	---	miR-let-7b expression was correlated with Mayo risk scores, IL-18 and ALP.	Qian et al. (2013)
miR-451a/miR-642-3p	---	Plasma exosomes	Up	---	Regulated the expression of the co-stimulatory molecules CD86 and CD80 in peripheral antigen-presenting cells	Tomiyama et al. (2017)
miR-197-3p/miR-505-3p	---	Serum	Down	---	As a clinical biomarker for PBC.	Ninomiya et al. (2013)
miR-122-5p/miR-141-3p	---	Serum	Up	---	As potential biomarkers of PBC.	Tan et al., (2014)
miR-26b-5p			Down			
miR-4311/miR-4714-3p	---	Serum	Down	---	Potential biomarkers for use in the development of treatment of patients with refractory PBC.	Sakamoto et al. (2016)
miR-122/miR-378			Up			
miR-155-5p	AKT3	PBMC	Down	MAPK/TCR/BCR signaling	A new disease marker of PBC.	Yang. et al. (2013)
miR-150-5p	---		Up			
**LncRNA**						
lncRNA NONHSAT250451.1	EGR1	PBMC	Up	Inflammation/immune activation/TCR signaling/NF-κB signaling/chemokine signaling	It is involved in inflammation, immune cell activation, TCR signaling pathway, etc., which may be related to the occurrence of PBC.	Xiang et al. (2019)
lncRNA AK053349	---	Peripheral blood CD8^+^ T cell and PBMC	Up	Autoimmunity and T lymphocyte activation	Targeted regulation of EGR1 may be involved in the occurrence of PBC.	Pang et al. (2009)
lncRNA XIST	Inflammatory cytokines	NK and CD4^+^ T lymphocytes	Up	Th1/Th17	Stimulated the secretion of IFN-γ, IL-17, TGF-β and ROR-γ T cells, and increase the proportion of Th1 and Th7 cells, led to the occurrence of PBC.	She (2020)
lncRNA H19	---	Liver tissue	Up	HSC activation and proliferation	Promoted HSC activation and proliferation, aggravate PBC.	(Li et al. (2018a); Liu et al. (2019); Li et al. (2020a))
**CircRNA**						
circ_402458/circ_087631/circ_406329	hsa-miR-522-3p/hsa-miR-943	Plasma	Up	Inflammation-related signaling	Candidate biomarkers for PBC.	Zheng et al. (2017a)
circ_407176/circ_082319			Down			

--- represents unknown.

PBMC, peripheral blood lymphocyte mononuclear cells; EMT, epithelial-mesenchymal transition; ALP, alkaline phosphatase; γ-GT, γ-glutamyl transpeptidase; TBIL, total bilirubin; TCR, T cell receptor; BCR, B cell receptor; NF-κB, nuclear factor-κB; MAPK, mitogen-activated protein kinase; HSC, hepatic stellate cell.

## MicroRNAs and Primary Biliary Cholangitis

### Broad Roles of MicroRNAs in Various Diseases

MiRNAs are evolutionarily conserved, non-coding small RNAs of 18–25 nucleotides in length. MiRNAs incompletely bind to complementary sequences in the 3′ untranslated region (3′UTR) of messenger RNA (mRNA) and regulate the expression of target genes at the post-transcriptional level by promoting the degradation of mRNA or repressing its translation (Tavasolian et al., 2018). MiRNAs can regulate about 90% of protein-coding genes and play important roles in various biological processes such as metabolism, cell differentiation, proliferation, apoptosis, and the maintenance of immune homeostasis (Alvarez-Garcia and Miska, 2005; Baltimore et al., 2008; Check Hayden, 2008). Disturbances in miRNAs expression profiles are associated with a variety of human diseases, including autoimmune diseases, such as systemic lupus erythematosus (SLE), PBC, and rheumatoid arthritis (RA) (Young et al., 2017; Wang et al., 2018a; Erice et al., 2018). Some of them have been proposed as non-invasive biomarkers of disease.

As shown in Table 1, various miRNAs were dysregulated in PBC. These miRNAs can regulate target genes of cytokines, oxidative stress, immunity and inflammation-related molecules, thereby participating in the pathogenesis or/and progression of PBC (Padgett et al., 2009; Qian et al., 2013; Liang et al., 2016; Erice et al., 2018; Song et al., 2018). Several published literatures have extensively explored the molecular mechanisms of differentially expressed miRNAs in PBC, particularly regarding their altering effects on inflammation and autoimmunity (Liang et al., 2016; Nakagawa et al., 2017; Erice et al., 2018).

### The Role of MiR-506 in the Pathogenesis of Primary Biliary Cholangitis

**FIGURE 2:**
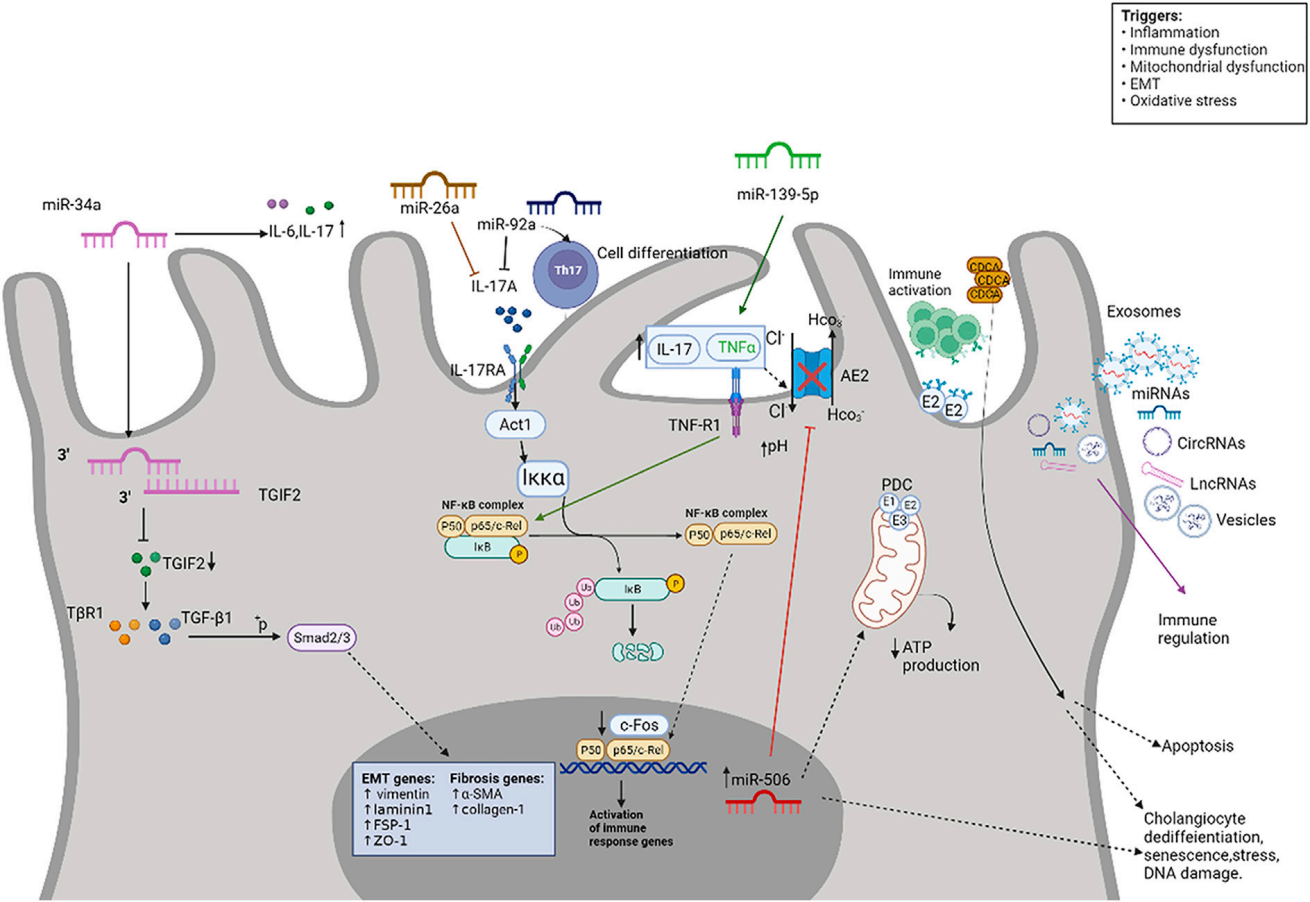
Signaling pathway of miRNAs in PBC. MiRNA are involved in regulating inflammation and autoimmunity. Some miRNAs are encapsulated in nanovesicles and exert critical effects on inflammatory and immune cells. miRNAs participate in PBC inflammation and autoimmune disorders primarily through NF-κB, TGF-β1, Th17, and so on.

Anion exchanger 2 (AE2) is essential in the maintenance of the protective bicarbonate- rich umbrella on the surface of BECs *via* regulation of biliary HCO_3_^−^ secretion, which shields BECs from noxious luminal bile acids (Rodrigues et al., 2018). Banales et al. (2012) demonstrated that miR-506 is upregulated in cholangiocytes from PBC patients, binds the 3′UTR region of AE2 mRNA and prevents protein translation, resulting in diminished AE2 activity and impaired biliary secretory functions. Given the putative pathogenic role of decreased AE2 in PBC, miR-506 may constitute a potential therapeutic target for this disease. Furthermore, miR-506 also regulates other genes involved in maintaining the integrity of bicarbonate umbrellas, the type III inositol 1,4,5-triphosphate receptor, an important regulator of calcium release from cholangiocytes (Ananthanarayanan et al., 2015; Erice et al., 2018). Under physiological conditions, acetylcholine increases the level of inositol triphosphate (InsP3) in cholangiocytes, resulting in an increase in the level of cytoplasmic Ca^2+^. Apical Cl^−^ secretion is further stimulated by the Ca^2+^ activated Cl^−^ channel transmembrane protein 16F (TMEM16A), ultimately leading to bicarbonate secretion through AE2 (Minagawa et al., 2007). The downregulation of InsP3R3 expression in cholangiocytes from PBC patients leads to decreased intracellular Ca^2+^ signaling and bicarbonate secretion, thereby triggering cholestasis (Shibao et al., 2003). InsP3R3 mRNA contains two highly conserved miR-506 binding sites, both of which are functional. In miR-506-overexpressed cholangiocytes, InsP3R3 mRNA and protein levels were reduced, resulting in a marked reduction in Ca^2+^ release from the endoplasmic reticulum and failure of bile secretion (Ananthanarayanan et al., 2015). *In vitro* data demonstrate that upregulation of proinflammatory, profibrotic, and senescent markers in miR-506-overexpressing cholangiocytes results in increased cellular stress and increased sensitivity to toxic hydrophobic bile acids (Erice et al., 2018). These results suggest a mechanistic link between epigenetic regulation, cellular damage, and immune dysregulation in PBC. Banales’ team further expiored the role of inflammatory factors, such as interleukins (IL)-1β, IL-6, IL-8, IL-12, IL-17, IL-18, tumor necrosis factor alpha (TNF-α) and interferon gamma (IFN-γ), transforming growth factor beta 1 (TGF-β1), estrogens (17β-estradiol, 17β-E2), bile acids [cholic acid (CA), UDCA and tauroursodeoxycholic (TUCA)] and other factors in regulating miR-506 expression in cholangiocytes and the role of miR-506 in cholangiocyte pathophysiology and immunomodulation in PBC. The result show different inflammatory factors enhance the expression of miR-506 in biliary epithelial cells. MiR-506 induces PBC-like features in cholangiocytes and promotes immune activation (Erice et al., 2018). Figure 2. Intriguingly, miR-506 is an X- linked miRNA localized to Xq27.3, which helps explain the possibility that females predominate in PBC disease, although whether this hypothesis applies to human remains to be demonstrated (Arora et al., 2013).

### Expression Profiles of MicroRNAs in Peripheral Blood and Liver Tissue of Primary Biliary Cholangitis Patients

Liang et al. (2016) used microarrays to identify 16 differentially expressed miRNAs (9 miRNAs upregulated and 7 miRNAs downregulated) in plasma from 3 PBC patients and 3 healthy controls. The most prominent finding was the downregulation of miR-92a, and the expression of miR-92a was negatively correlated with the Th17 cell population. Furthermore, the expression of miR-92a was colocalized with IL-17A in peripheral blood mononuclear cells (PBMCs) of patients, implying a direct regulation of IL-17A by miR-92a. Notably, studies have shown that Th17 cells and the IL-17 secreted by the cells may induce epithelial-mesenchymal transition (EMT) in intrahepatic bile ducts through IL-17A-IL-17RA-Act1 activation of the NF-κB pathway, and participate in the progression of PBC disease (Huang et al., 2016). Figure 2. Domestic studies have found that there are differences in the expression of miR-26a in initial CD4^+^ T cells, memory CD4^+^ T cells and effector CD4^+^ T cells of PBC patients, and no expression in Jurkat cells (human T cell line). The authors then cultured Th17 cells (derived from human CD4^+^ T cells) and transfected pre-miR-26a and anti-miR-26a, respectively. The results showed that compared with the normal control, the cells transfected with pre-miR-26a significantly decreased IL-17, significantly decreased the activities of NF-κB and AP-1, and induced increased apoptosis after activation; transfected with anti-miR-26a cells, IL-17 mRNA, NF-κB and AP-1 activities were significantly increased, cell proliferation activity increased, and apoptosis induced by activation decreased. The luciferase reporter further demonstrated that miRNA-26a could indeed regulate IL-17. Therefore, this study shows that miRNA-26a regulates the function of Thl7 cells by affecting apoptosis and cell proliferation activities induced by IL-17 activation (Tang, 2009). Figure 2. The above studies demonstrate that miRNAs may play a significant role in the fibrosis of bile duct epithelial cells in PBC patients through IL-17.

Pan et al. (2021) found that miR-34a was significantly overexpressed in the serum of PBC patients (30 PBC vs.30 controls). miR-34a upregulation inhibits proliferation of intrahepatic bile duct epithelium and increases mesenchymal markers zonula occluden-1 (ZO-1), laminin 1, vimentin and fibroblast-specific protein 1 (FSP-1) and expression of fibrotic markers α-SMA, and collagen I. The authors further demonstrated that miR-34a targets TGIF2 to induce EMT and fibrosis in intrahepatic bile duct epithelium through the TGF-β1/smad pathway (Pan et al., 2021). Figure 2. MiR-139-5p was significantly downregulated in clinically advanced PBC compared to control. *In situ* hybridization and RT-qPCR showed that the expression of miR-139-5p in lymphocytes of PBC patients was higher than that of lymphocytes of other liver diseases (chronic viral hepatitis and autoimmune hepatitis), while miR-139-5p in hepatocytes of PBC was lower than that in other liver disease, suggesting that lymphocytes are the main source of miR-139-5p. *In vitro* studies showed that with the upregulation of miR-139-5p, the level of TNF-α in the cell supernatant was significantly increased, the transcription of the c-FOS gene was inhibited, and the NF-κB signaling pathway was finally activated. Activation of NF-κB further induces the production of TNF-α, and increased levels of TNF-α may accelerate bile duct injury through positive feedback. The authors revealed a novel inflammation-regulatory mechanism between TNF-α and c-FOS transcription through miR-139-5p in the NF-κB pathway (Katsumi et al., 2016). Figure 2. Therefore, miR-139-5p could become not only a biomarker of disease progression, but also a novel therapeutic target for patients with progressive PBC. In addition, the expression of miR-155 was enhanced in PBMCs and liver tissues of PBC patients, accompanied by vitamin D receptor (VDR) mRNA and protein, cytokine signaling inhibitor 1 (SOCS1) protein expression decreased, indicating that the decreased VDR expression may lead to the dysregulation of the negative feedback loop through the VDR-miRNA155-SOCS1 pathway, thereby triggering a sustained inflammatory response (Kempinska-Podhorodecka et al., 2017). Padgett et al. (2009) analyzed liver tissue from 6 patients with end-stage PBC and 5 control subjects using a miRNA microarray platform. The results showed that 35 differentially expressed miRNAs (11 upregulated and 24 downregulated) were identified in the liver tissue of PBC patients compared with the liver tissue of normal controls. The targets they predicted were associated with the regulation of cell proliferation, apoptosis, inflammation, oxidative stress, and metabolism. According to the above studies, we can conclude that miRNAs may be derived from various immune cells and play a significant effect in the occurrence and development of PBC by inflammatory factors and immune-inflammatory pathways (such as TGF-β1/smad, TCR, NF-κB, and so on). Subsequent studies on miRNAs and PBC can start from immune cells and immune-inflammation-related pathways. Subsequent studies on miRNAs and PBC can start from immune cells (not only B cells, T cells, but also Th17 cells, Treg cells, macrophages, etc.), inflammatory factors and immune-inflammation-related pathways.

### MicroRNAs and Therapeutic Targets for Primary Biliary Cholangitis

Nakagawa et al. (2017) examined total RNAs of CD4^+^ T cells from 6 PBC patients and 6 healthy controls using miRNA microarrays. The authors found that the expression levels of 16 miRNAs were significantly different in PBC patients compared with healthy controls (*p* < 0.05, fold change >1.2). Among them, five of these miRNAs were significantly downregulated in PBC patients by qRT-PCR. The integral analysis of miRNA and mRNA identified four significantly downregulated miRNAs (miR-181a, miR-181b, miR-374b, and miR-425) related to the T-cell receptor (TCR) signaling pathway in CD4^+^ T cells of PBC. N-Ras, a regulator of the TCR signaling pathway, was found to be targeted by all four identified miRNAs. In addition, *in vitro* assays confirmed that decreased miR-425 strongly induced inflammatory cytokines (IL-2 and IFN-γ) *via* N-Ras upregulation in the TCR signaling pathway. Therefore, the restoration of decreased miR-425 or the suppression of N-Ras may be promising for an immunotherapeutic strategy against PBC.

The authors believe that miR-122 is the most noteworthy therapeutic target for PBC. MiR-122 is a conserved liver-specific miRNA, accounting for 70% of total liver miRNAs (Chang et al., 2008). Multiple studies have shown that miR-122 plays a key role in lipid metabolism (Esau et al., 2006), cell differentiation (Kim et al., 2011), liver polyploidy (Hsu et al., 2016), hepatitis C virus replication (Luna et al., 2015), acetaminophen toxicity (Chowdhary et al., 2017; Yang et al., 2021), liver fibrosis in innate immunity of hepatocytes (Xu et al., 2019). Decreased miR-122 expression was found in HCV-negative liver cancer and was associated with metastasis in hepatocellular carcinoma (HCC) patients (Coulouarn et al., 2009; Tsai et al., 2009).

Tan et al. (2014) analyzed miRNA expression by Illumina sequencing of serum samples from 3 PBC patients and 3 controls and assessed the expression of selected miRNAs in a screened group (*n* = 40) by qRT-PCR. A logistic regression model was then constructed using the training cohort (*n* = 192) and validated with another cohort (*n* = 142). The results showed that serum miR-122-5p levels were elevated in PBC patients. Compared with ALP and ANA, the miRNA panel (hsa-miR-122-5p, hsa-miR-141-3p, and hsa-miR-26b-5p) was a more sensitive and specific biomarker for PBC (Tan et al., 2014). Circulating miR-122 levels have been reported to correlate with liver histological stage, inflammation grade, and ALT activity (Lewis and Jopling, 2010; Zhang et al., 2010; Hu et al., 2012; Wang et al., 2012; Iino et al., 2013). MiR-122 expression was significantly decreased in a carbon tetrachloride (CCl4)-induced mouse model of liver fibrosis and HCC (Li et al., 2013; Hayes and Chayama, 2016). Similarly, Padgett et al. (2009) used the miRNA array platform to analyze the liver tissue of 6 patients with end-stage PBC and 5 control subjects, and the results showed that miR-122 was also downregulated in the liver tissue of PBC patients. This is an interesting result. It means that the liver may overexpress miR-122 compensatory in the early stage of PBC. However, most of miR-122 reaches the peripheral circulation, which increases miR-122 in peripheral circulation in PBC patients; or in PBC patients, other tissues, organs or cells abnormally express miR-122 in addition to the liver. The above-mentioned known immune cells and inflammatory factors play an important role in the occurrence and development of PBC. Therefore, the authors speculate that in the peripheral blood of PBC patients, various immune cells are activated (such as T cells, B cells, Th cells, Treg cells, macrophages, etc.) abnormally expressing miR-122 increases the level of miR-122 in serum/plasma. MiR-122 is a negative regulator of liver fibrosis. Li et al. (2013) found that miR-122 inhibited the proliferation and activation of Lx2 in hepatic stellate cells (HSC) by targeting P4HA1, regulating collagen production, and inhibiting liver fibrosis.

Tsai et al. (2012) generated a mutant mouse strain with a germline deletion of *Mir122a* using homologous recombination (*Mir122a* ^*−/−*^). Histological examination of the livers of Mir122a−/− mice revealed extensive lipid accumulation and reduced glycogen storage, as well as inflammation and fibrosis, compared with WT controls. A strong positive reaction to the anti-F4/80 antibody, which is specific for mouse macrophages and monocytes, was detected in the Mir122a−/− livers. Teng et al. (2020) also found that knockout of miR-122 in mouse liver developed spontaneous liver fibrosis. Therefore, the authors speculate that miR-122 may be a novel gene therapy strategy for patients with advanced PBC, especially targeting the liver. Whether the anti-fibrotic mechanism of miR-122 is related to its regulation of hepatic fatty acid and cholesterol synthesis still needs to be further explored (Esau et al., 2006; Naderi et al., 2017). In addition, due to the ability of miR-122 to enhance HCV replication, vigilance and monitoring of HCV infection should be exercised when using miR-122 (Luna et al., 2015). In the early stage of PBC, the main lesions are chronic inflammation and fibrosis of the intrahepatic bile ducts. Whether miR-122 can regulate the proliferation and apoptosis of intrahepatic bile duct cells has not been reported in the literature. This is also the direction our research group is working on.

### Immunomodulatory Role of Exosomal MicroRNAs in Primary Biliary Cholangitis

Extracellular vesicles (EVs) are nano- or micro-lipid bilayer spheres produced by different cells. They are released into the extracellular space where they participate in intercellular communications. They are also found in bile and contain miRNAs (Li et al., 2014). Several nanovesicle-delivered miRNAs have been identified that are specifically expressed in PBC and play a modifying role in inflammation and autoimmunity (Olaizola et al., 2018). Exosomes are small vesicles formed by budding from endosomal membrane and released to extracellular by fusion with plasma membrane, which are important mediators of intercellular communication (Hirsova et al., 2016a; Martínez and Andriantsitohaina, 2017). Recent studies have reported that exosomes-mediated transfer of ncRNAs, proteins and lipids are associated with a variety of human diseases, including liver disease (Bala et al., 2012; Hirsova et al., 2016a; Debabrata and Mukhopadhyay, 2017; Pan et al., 2017; Szabo and Momen-Heravi, 2017). Hepatic epithelial cells, including cholangiocytes and hepatocytes, are exosomes-releasing cells (Masyuk et al., 2010; Masyuk et al., 2013; Hirsova et al., 2016b; Worst et al., 2017). Tomiyama et al. (2017) found plasma-derived exosomal miR-451a and miR-642a-3p were increased in PBC patients compared with healthy controls, and could regulate the expression of the co-stimulatory molecules such as CD86 and CD80 in peripheral antigen-presenting cells. Figure 2. In conclusion, accumulating evidence points to the critical role of miRNAs in regulating inflammation and autoimmunity, and many mature miRNAs are expected to be candidate biomarkers and therapeutic targets for PBC (Ninomiya et al., 2013; Qian et al., 2013; Yang, 2013; Katsushima et al., 2014; Tan et al., 2014; Sakamoto et al., 2016).

## Long Non-Coding RNA and Primary Biliary Cholangitis

### Expression Profiles of Long Non-Coding RNAs in Autoimmune Diseases

LncRNAs are highly conserved RNA sequences >200 nucleotides in length that can epigenetically regulate gene expression and broadly affect cellular biological processes (Mercer et al., 2009; Ponting et al., 2009). Published literature on lncRNA have been focused on cancer (Chan and Tay, 2018; Mitobe et al., 2018; Tian et al., 2018), and studies on lncRNA in innate immunity are relatively scarce, accounting for only about 4% of all lncRNA papers (Robinson et al., 2020). With increasing interest of lncRNA in autoimmune diseases, it has been found that different autoimmune diseases (including PBC) have specific lncRNA expression profiles in different cells and tissues (Zhang et al., 2013; Sigdel et al., 2015; Hur et al., 2019; Liang et al., 2019). Previous studies have shown that the lncRNA AK053349 is highly expressed in CD8^+^ T cells and is associated with autoimmunity and T lymphocyte activation (Pang et al., 2009). Zhang et al. (2013) wrote in his doctoral dissertation that the lncRNA AK053349 was increased in PBMC of PBC patients and positively correlated with the Mayo risk score, emphasizing its potential relevance to the pathogenesis of PBC and worthy of further study (Zhang et al., 2013). Another recent domestic study (She, 2020) found that the expression level of lncRNA XIST in NK cells and CD4^+^ T lymphocytes of PBC patients was significantly higher than that of healthy controls, and could be clearly located in the nucleus. The high expression of lncRNA XIST in Naïve CD4^+^ T cells of PBC patients can promote the proliferation of Naïve CD4^+^ T cells, stimulate the secretion of IFN-γ, IL-17, TGF-β and ROR-γ T cells, and increase the proportion of Th1 and Th7 cells, which led to the occurrence of PBC. This further supports the critical role of immune cells and inflammatory factors, especially IL-17 in PBC disease.

### Exosomal Long Non-Coding RNA H19 Promotes Bile Duct Proliferation and Liver Fibrosis

**FIGURE 3:**
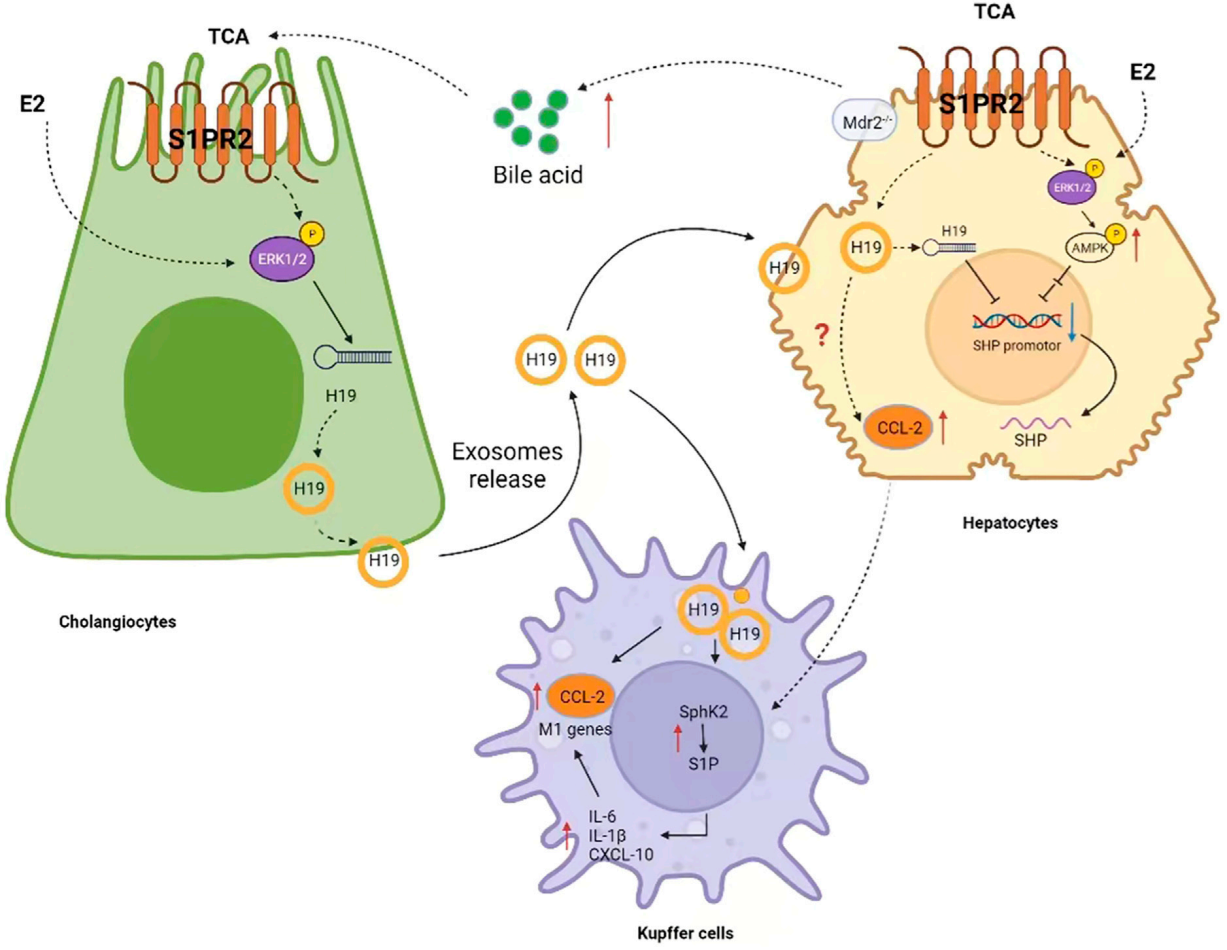
Molecular mechanism of lncRNAH19 promoting cholestasis and activating macrophages.

Huiping Zhou et al. (Li et al., 2017) used Mdr2^−/−^ mice as an animal model of cholestatic biliary disease. Their study showed that H19 expression was significantly increased in the liver and bile duct cells of female Mdr^2−/−^ mice compared with male Mdr2^−/−^ mice, and that abnormal H19 expression was associated with the severity of biliary fibrosis in female Mdr2^−/−^ mice. Knockdown of H19 alleviate cholestatic liver injury in female Mdr2^−/−^ mice. Furthermore, both Taurocholic acid (TCA) and Estradiol 2 (E2) upregulated the expression of H19, but there was no superposition or synergy. The role of H19 in cholestatic injury in female Mdr2^−/−^ mice may be related to extracellular regulated protein kinase 1/2 (ERK1/2) signaling pathway. In a follow up study, Huiping Zhou’s team further demonstrated that exosomal H19 is derived from bile duct cells and transferred to hepatocytes, inhibit the expression of small heterodimeric partner (SHP) in hepatocytes and promote cholestatic injury (Li et al., 2018a). Figure 3. To further determine the effect of H19 expression in the progression of liver fibrosis, the authors examined the expression levels of hepatic H19, Ck19, and fibrosis marker genes (Acta2, Loxl2, and Collagen 1) in both male and female 2-week bile duct ligation (BDL) mice and in 100 day old female Mdr2^−/−^ mice and perform a linear analysis. The results showed that the expression level of hepatic H19 was significantly positively correlated with Ck19 and fibrosis marker genes. Similarly, in primary sclerosing cholangitis (PSC) and PBC patients, the hepatic mRNA levels of H19, CK19, and fibrosis marker genes were all increased. In BDL mice with H19 knockout (H19KO-BDL), collagen deposition and α-SMA-positive fibroblast distribution near the bile ducts of mice were significantly reduced. H19 deficiency also significantly alleviated serum AST, ALT, ALP, and total bile acid (TBA) levels induced by BDL. H19 deficiency in DKO (Mdr2 and H19 double knockout) mice significantly reduced bile duct proliferation, immune cell infiltration and fibrosis in the periportal area. Next, the authors isolated exosomes from H19-enriched and H19-free (control) cholangiocytel culture medium and injected mice *via* tail vein. The results showed that bile duct proliferation, hepatic inflammation, collagen deposition, and fibroblast activation were more severe in mice treated with H19-enriched exosomes when compared to those treated with control exosomes. This effect is related to the promotion of hepatic stellate cell (HSC) activation and proliferation by exosomal H19 (Liu et al., 2019). Furthermore, cholangiocyte-derived exosomal H19 promotes macrophage activation, differentiation, chemotaxis and liver inflammation through the CCL-2/CCR-2 signaling pathway. Figure 3. H19-deficiency ameliorates the liver cholestasis and macrophage activation in both BDL and Mdr2^−/−^ Mice (Li et al., 2020a). Thus, exosomal H19 represents a noninvasive biomarker and potential therapeutic target for cholestatic disease. Notably, Mdr2^−/−^ mice are actually a suitable model for PSC and not an animal model for PBC disease. What role lncRNA H19 plays in PBC disease and whether the molecular mechanism is the same remains to be discussed.

## Circular RNAs and Primary Biliary Cholangitis

CircRNA is an endogenous non-coding RNA, the most representative characteristic of which is the covalently closed RNA circle (Salzman, 2016; Ouyang et al., 2017). Circularization results in resistance and high stability of RNA to exonuclease-mediated degradation. In addition, circRNAs are abundant and evolutionarily conserved in the cytoplasm. These properties make circRNAs potentially more suitable as clinical biomarkers than other types of RNAs (Jeck and Sharpless, 2014; Lasda and Parker, 2014; Li et al., 2015). Most importantly, the expression profiles of circRNAs are specific in different cell types and can generally be found in peripheral blood, exosomes, and tissues. Due to their various forms of epigenetic modifications, circRNAs play important roles in various diseases, such as cancer, neurologic disorders and cardiovascular diseases (Zhao et al., 2017; Yang et al., 2018a; Ojha et al., 2018; Sheng et al., 2018). Ma et al. (2019) showed that circARSP91 promotes cancer immune surveillance by regulating NK cells in liver cancer, suggesting a key role of circRNAs in tumor immunity. Furthermore, circRNA Malat-1 is thought to act as a key regulator of alloimmune rejection by promoting dendritic cell-induced T cell exhaustion and regulatory T cell generation, suggesting a critical role for circRNAs in adaptive immunity (Zhang et al., 2018). In conclusion, circRNAs play key roles not only in innate immunity but also in adaptive immunity.

In recent years, accumulated studies have shown that circRNAs are closely related to the occurrence and development of autoimmune diseases (Yang et al., 2018b), including SLE (Li et al., 2018b; Wang et al., 2018b), RA (Zheng et al., 2017b), PBC (Zheng et al., 2017a), etc. Zheng et al. (2017a) used microarray to identify 22 aberrantly expressed circRNAs (18 upregulated, 4 downregulated) in the plasma of PBC patients. Notably, PBC patients who did not receive UDCA had higher levels of hsa-circ-402458 than those who received UDCA. Hsa-circ-402458 may target hsa-miR-943 and hsa-miR-522-3p. For miR-522-3p, it may be an effective target for regulating chronic inflammatory diseases. Therefore, the authors speculate that hsa-circ-402458 may act as a miRNA sponge to regulate inflammation-related pathways, thereby promoting the development of PBC. Unfortunately, there are few studies on the relationship between circRNAs and PBC, and the molecular mechanism behind the regulation of circRNAs in PBC disease is still unclear. Whether circRNAs, like miRNAs and/or lncRNAs, play a role in PBC through signaling pathways such as TGF-β, NF-κB, TLR, TCR and oxidative stress, remains to be further explored.

## Conclusion and Future Directions

Studies on ncRNAs in human biology have gained much interest in the scientific world in recent years (Kapoor et al., 2021; Makkos et al., 2021; Wang et al., 2021). The role of ncRNAs in immune regulation, inflammation and autoimmunity can be of significant translational implication in medicine. Although specific expression profiles of miRNAs, lnRNAs, and cirRNAs have been well-documented in the literature (Padgett et al., 2009; Sigdel et al., 2015; Zheng et al., 2017a), the underlying mechanisms of ncRNAs in the development of PBC is unclear and may involve autoimmune regulatory pathways such as TGF-B1, NF-kB, Th17, and TCR. Furthermore, the effects of ncRNAs in oxidative stress, apoptosis, immune cells homing, and others in PBC are also confounding factors. Further studies including a combination of wet-bench studies and the use of bioinformatics tools (Li et al., 2020b; Zhu and Leung, 2021) to discover the target gene networks of non-coding RNAs are necessary to decipher the mechanistic role of ncRNAs in the pathogenesis of PBC and their potential application as diagnostic markers and/or therapeutic checkpoints.
